# Characterization of Chlorophyll Degradation Genes Reveals Gene Cluster HuSGR2 and HuSGR3 Promoting Chlorophyll Degradation in Pitaya Peel

**DOI:** 10.3390/genes17040427

**Published:** 2026-04-05

**Authors:** Wenting Wu, Tian Yang, Yun Lan, Zeyu Zheng, Xiaoying Ye, Meibing Ma, Canbin Chen, Fangfang Xie

**Affiliations:** 1 State Key Laboratory for Conservation and Utilization of Subtropical Agro-Bioresources, Guangxi Key Laboratory for Agro-Environment and Agro-Product Safety, College of Agriculture, Guangxi University, Nanning 530004, China; wwenting2022@163.com (W.W.);; 2 Horticulture Research Institute, Guangxi Academy of Agricultural Sciences, Nanning 530007, China; 3 Guangxi Zhuang Autonomous Region Fusui Breeding Livestock Farm, Chongzuo 532113, China

**Keywords:** pitaya, chlorophyll degradation, SGR protein, transient expression

## Abstract

Background: Chlorophyll degradation is a characteristic sign of fruit ripening. However, the chlorophyll degradation pathway during pitaya fruit development remains unexplored. Methods and Results: Here, chlorophyll contents showed a downward trend across the five developmental stages of ‘Jindu No.1’ pitaya peels. Based on the pitaya genome data, twenty chlorophyll degradation genes were identified, including two *NYCs*, three *CLHs*, five *SGRs*, six *PAOs*, and four *RCCRs*, spread across eight pitaya chromosomes. In addition, their phylogenetic relationships, conserved motifs, and domains were analyzed using homologous genes from beet and Arabidopsis species. Transcriptomic data and RT-qPCR analyses of these genes suggested that three *HuSGRs* demonstrated a significant upward trend during pitaya peel maturation. Indeed, the *HuSGR1* has the complete gene structure, including the chloroplast transit peptide, SGR domain, and variable C-terminal region. However, *HuSGR2* and *HuSGR3* contained the N- and C-terminal sequences, respectively, of *HuSGR1*. They were separated by a 690 bp distance on chromosome 8, forming a gene cluster. Overexpressed *HuSGR2* or *HuSGR3* alone resulted in a significant decrease in chlorophyll contents in tobacco leaves. Notably, a more obvious reduction of chlorophyll contents was observed when overexpressing them together. Conclusions: Our results show that *HuSGR2* and *HuSGR3* were involved in accelerating the chlorophyll degradation process, providing new insights into the molecular basis of color formation in pitaya peels.

## 1. Introduction

Pitaya is a perennial climbing fruit crop pertaining to the *Hylocereus* and *Seleniereus* genus of the Cactaceae family. It is native to the tropical and subtropical regions of Central America and widely cultivated in many countries, such as China, Mexico, Peru, Colombia, and Venezuela. Due to its rich nutrition, characteristic shape, attractive color, medicinal use, and industrial values, pitaya gained more attention and became more popular among planters, consumers, and researchers [1]. In the market, three common pitaya types with different colors are available: white flesh with red peel pitayas (*Hylocereus undatus*), red flesh with red peel pitayas (*H. polyrhizus*/*H. monacanthus*), and white flesh with yellow peel pitayas (*S. megalanthus*).

Fruit color is a genetic trait and represents one of the key indicators of crop fruit quality. During fruit development and maturation stages, fruit peels generally undergo a marked color transition, progressing from an initial green stage to mature red or yellow pigmentation. Several studies have indicated that the shift from green to red or yellow coloration in peel is accompanied not only by chlorophyll degradation, but also by carotenoid biosynthesis in tomato and banana [2,3], anthocyanin biosynthesis in apple and litchi [4,5], and betalain biosynthesis in pitaya [6]. Thus, chlorophyll degradation plays an important role in the loss of green color and the development of yellow and red hues during peel ripening.

To date, the chlorophyll degradation pathway has been extensively studied in numerous fruit crops, including litchi [4], mango [7], and citrus [8], and multiple enzymes have been verified to participate in this pathway. NON-YELLOW COLORING1 (NYC1) and NYC1-LIKE (NOL) encode chlorophyll *b* reductase belonging to the short-chain dehydrogenase/reductase family. They play important roles in the conversion of chlorophyll *b* to chlorophyll *a*, a process known as chlorophyll cycling. Functionally, *NYC1* has been characterized in banana, rice, and Arabidopsis, where a decrease in *NYC1* expression gives rise to a stay-green phenotype with high chlorophyll contents [2,9,10]. Thereafter, a hydrolase enzyme named chlorophyllase (CLH) catalyzes the chlorophyll *a*/*b* to chlorophyllide *a*/*b*, initiating chlorophyll degradation. Data from *Paeonia suffruticosa* and bottle gourd suggested that silencing the *CLH* gene promoted chlorophyll *a* accumulation [11,12], while overexpression of *MdCLH1* diminished the chlorophyll *a* content in apple leaves [13].

Interestingly, chlorophyll *a* degradation was also carried out by a magnesium dechelatase named STAY-GREENs (SGRs), which removes the magnesium from chlorophyll *a* and turns it into pheophytin *a* and pheophorbide *a* [14]. In higher plants, the SGR superfamily is classified into the SGR subfamily and the SGR-like subfamily (SGRL). Overexpression of *Pisum sativum SGR* and *SGRL* separately in tobacco leaves leads to a significant reduction in chlorophyll content, and *PsSGR* exhibited a more obvious de-greening phenotype [15]. The same results were obtained when *Litchi chinensis SGR* and *SGRL* were overexpressed in tobacco leaves [4]. Finally, pheophorbide *a* is oxygenolytically converted to a transient intermediate named red chlorophyll catabolite (RCC) by pheophorbide *a* oxygenase (PAO), then produces primary fluorescent chlorophyll catabolites (pFCC) utilizing red chlorophyll catabolite reductase (RCCR), and stored in vacuoles. Indeed, both the Arabidopsis *acd1*/*pao1* and *acd2-2*/*rccr* mutant leaves demonstrated a higher chlorophyll content than the wild-type Arabidopsis [16,17]. In rice, knockdown of *OsPAO* results in the production of pheophorbide *a* at the seedling stage, while knockdown of *OsRCCR1* results in a deep red color (probably RCC) in older leaves [18]. Although the chlorophyll degradation pathway has made great progress in multiple fruits, it remains poorly characterized in pitaya.

In pitaya peels, betalain biosynthesis is accompanied by chlorophyll degradation during fruit development and maturation. Many researchers focused on clarifying the mechanisms of betalain biosynthesis and regulation [6,19], but the mechanisms of chlorophyll degradation remain unclear. In this study, chlorophyll contents during fruit development and maturation were determined in pitaya peels. The chlorophyll degradation genes, i.e., *HuNYC1*, *HuCLHs*, *HuSGRs*, *HuSGRLs*, *HuPAOs*, and *HuRCCRs*, were identified utilizing the pitaya genome. Further analyses, including chromosome localization, conserved domains and motifs, synteny, and expression levels, were performed. Among them, the functional roles of the critical genes *HuSGR2* and *HuSGR3* in chlorophyll degradation were investigated. Our findings aim to provide a thorough understanding of chlorophyll degradation in pitaya peels.

## 2. Materials and Methods

### 2.1. Plant Material

‘Jingdu No.1’ pitaya (‘JD1’, *H. monacanthus*) is a popular cultivar grown in Guangxi, China, with red peel and red flesh features. Pitaya materials used in this study were collected from the orchard of Jinxiufengnian (Wuming District, Nanning City, Guangxi Province, China). Due to the changes in peel coloration, the pitaya peels of ‘JD1’ were collected on the 15th (light green), 19th (green), 23rd (dark green), 27th (coloring), and 31st (red) days after pollination from different plants with three biological repeats. Tobacco (*Nicotiana benthamiana*) plants were grown in a greenhouse under a 16 h/8 h day/night cycle at 25 °C, and used for the transient overexpression assay. All samples were immediately frozen in liquid nitrogen and stored at −80 °C until needed.

### 2.2. Measurement of Chlorophyll Contents

Chlorophyll *a* and *b* were extracted and measured based on a previously described method [19] with minor modifications. A total of 0.5 g freeze-dried pitaya peel was mixed with 5 mL of an 80% (*v*/*v*) acetone solution, then sonicated for 10 min in an ultrasonic cleaner (SB25-12DT, Ningbo, China). Then, the mixtures were stilled in the dark at room temperature for 1 h, and centrifuged at 5000 revolutions per minute for 10 min to collect the supernatants. The supernatants were measured through spectrophotometry (Innite M200, Tecan Co., Ltd., Shanghai, China) at 645 nm and 663 nm. All determinations were performed in three biological replicates.

### 2.3. Identification of Pitaya Chlorophyll Degradation-Related Genes

The amino acid sequences of AtNYCs, AtCLHs, AtSGRs, AtPAOs, and AtRCCR were obtained from the Arabidopsis Genome Database (Gene IDs and protein sequences listed in Table S1, https://www.arabidopsis.org/). They were utilized as queries to identify chlorophyll degradation-related genes from the beet (GenBank number, GCA_026745355.1) and pitaya genomes (http://www.pitayagenomic.com/) via the Several Sequences to a Big File program of TBtools-II software with e-value < 1 × 10^−5^ [20,21]. The resulting protein sequences were subsequently analyzed using the Conserved Domain Database (https://www.ncbi.nlm.nih.gov/, accessed on 29 March 2025) to verify the presence of their conserved domains. All the identified chlorophyll degradation genes in beet and pitaya were listed in Table S1. The molecular weights (MWs) and theoretical isoelectric points (pIs) of these proteins were analyzed on the ExPASy Server (https://web.expasy.org/protparam/, accessed on 29 March 2025).

### 2.4. Chromosomal Locations and Synteny Analyses

The GFF file containing the chromosomal location information of chlorophyll degradation genes was obtained from the pitaya genome database [20]. The MapChart software (version 2.32) was used to draw the locations of chlorophyll degradation genes on pitaya chromosomes based on their physical positions [22]. The collinearity relationships of chlorophyll degradation genes among pitaya, beet, and Arabidopsis species were illustrated by the One Step MCScanX-Super Fast program of TBtools-II software.

### 2.5. Phylogenetic, Conserved Motif, and Domain Analyses

Phylogenetic trees of pitaya, beet, and Arabidopsis NYC, CLH, SGR, PAO, and RCCR proteins were constructed individually using the maximum likelihood method (ML) in MEGA 7 with 1000 bootstrap replicates (https://www.megasoftware.net/). The conserved motifs of complete amino acid sequences were analyzed by Multiple Em for Motif Elicitation (https://meme-suite.org/, accessed on 30 March 2025) with five or ten motifs. The conserved domains of proteins were analyzed using the Conserved Domain Database tool in NCBI (https://www.ncbi.nlm.nih.gov/cdd, accessed on 30 March 2025). The phylogenetic tree, motif compositions, and conserved domains of pitaya, beet, and Arabidopsis NYC, CLH, SGR, PAO, and RCCR proteins were visualized individually using the Gene Structure View program of TBtools-II.

### 2.6. Gene Expression Analyses and Cloning

Transcriptome data (NCBI BioProject number, PRJNA1444569) from three developmental stages (19th, 23rd, and 27th) of ‘JD1’ pitaya peels and flesh, with three biological replicates, were used to draw the transcript abundance of chlorophyll degradation genes using the HeatMap program of TBtools-II software (Table S2). Total RNA from pitaya peels was isolated following the manufacturer’s protocol of the EASYspin Plus Complex Plant RNA Kit (RN53) (Aidlab Biotechnology, Beijing, China). The cDNA for each sample was synthesized based on the protocol for the PrimeScript™ RT Reagent Kit with gDNA Eraser (TaKaRa, Shiga, Japan). RT-qPCR was conducted on an ABI 7500 Real-Time PCR System (Thermo Fisher Scientific, Waltham, MA, USA) with the ChamQ Universal SYBR qPCR Master Mix (Vazyme, Nanjing, China) and specific primers (Table S3). The specific primers were designed by the Primer 3-BLAST tool of NCBI (https://www.ncbi.nlm.nih.gov/tools/primer-blast/, accessed on 2 April 2025). *Actin (1)* was employed as an internal control for gene expression analyses in pitaya [23]. The relative expression levels of each candidate gene were calculated using the comparative Ct method [24]. All experiments were repeated in technical replicates.

The full-length coding sequences (CDSs) of *HuSGR2* and *HuSGR3* were cloned with I-5^TM^ 2× High-Fidelity Master Mix (MCLAB, San Francisco, CA, USA) and specific primers (Table S3). Alignment of the protein sequences was performed by the MUSCLE method of MEGA 7 and visualized by GeneDoc software (version 2).

### 2.7. The Transient Overexpression of HuSGRs in N. benthamiana

The transient overexpression assay in *N. benthamiana* was carried out according to a previously described method [25]. The CDSs of *HuSGR2* and *HuSGR3* were respectively inserted into the pEAQ vector to create pEAQ-HuSGR2 and pEAQ-HuSGR3 fusion constructs (primers listed in Table S3), which were separately transformed into *Agrobacterium tumefaciens* strain GV3101 and then infiltrated into *N. benthamiana* leaves. Five days after infiltration, *N. benthamiana* leaves with three biological replicates were immediately frozen in liquid nitrogen and stored at −80 °C for chlorophyll measurement and gene expression analyses.

### 2.8. Statistical Analyses

The comparisons between different groups were performed using a one-way analysis of variance (One-way ANOVA) with the Duncan test (*p* < 0.05) and the homogeneity of variance test in SPSS 25 software (IBM, Chicago, IL, USA). The results were presented as mean ± standard error, with lowercase letters indicating significance values.

## 3. Results

### 3.1. Chlorophyll Changes in ‘JD1’ Pitaya Peels

**Figure 1:**
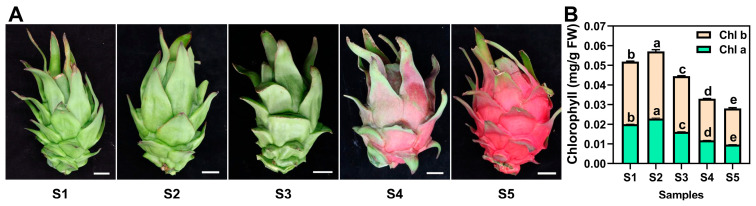
Phenotype analyses of five developmental stages of ‘JD1’ pitaya peels. (**A**), Images of five different developmental stages of ‘JD1’ pitaya peels. S1 to S5 represent the fruits collected on the 15th, 19th, 23rd, 27th, and 31st days after pollination, respectively. Bars indicate 2 cm. (**B**), Chlorophyll *a* and *b* contents for the five developmental stages in ‘JD1’ pitaya peels. Data represent the mean ± S.E. of three biological replicates. Lowercase letters indicate comparisons between groups using one-way ANOVA (Duncan test, *p* < 0.05).

To investigate the changes in chlorophyll metabolism during the developmental stages of pitaya peels, we collected peels at five different stages (termed S1 to S5) of ‘JD1’ pitaya, with three biological replicates. The results showed that the peel color changed from light green to dark green to red during its development and maturation (Figure 1A). For ‘JD1’ pitaya peels, the contents of chlorophyll *a* and *b* exhibited a gradual increase from S1 to S2, followed by a significant decrease from S2 to S5 (Figure 1B). The results suggested that total chlorophyll increased early and then declined during pitaya development and maturation.

### 3.2. Identification of Chlorophyll Degradation-Related Genes from H. undatus

The pitaya and beet chlorophyll degradation-related genes were identified by aligning the pitaya and beet genomes with the AtNYCs, AtCLHs, AtSGRs, AtPAOs, and AtRCCR protein sequences using the BLASTP program in TBtools software. A total of twenty chlorophyll degradation-related genes were obtained from the pitaya genome, including two *NYCs* (*HuNYC1*, and *HuNYC2*), three *CLHs* (*HuCLH1*, *HuCLH2*, and *HuCLH3*), five *SGRs* (*HuSGR1*, *HuSGR2*, *HuSGR3*, *HuSGRL1*, and *HuSGRL2*), six *PAOs* (*HuPAO1*, *HuPAO2*, *HuPAO3*, *HuPAO4*, *HuPAO5*, and *HuPAO6*), and four *RCCRs* (*HuRCCR1*, *HuRCCR2*, *HuRCCR3*, and *HuRCCR4*) (Table S1). Meanwhile, twelve chlorophyll degradation-related genes were obtained from the beet genome, including two *NYCs* (*BvNYC1* and *BvNYC2*), two *CLHs* (*BvCLH1* and *BvCLH*), two *SGRs* (*BvSGR1* and *BvSGRL*), five *PAOs* (*BvPAO1*, *BvPAO2*, *BvPAO3*, *BvPAO4*, and *BvPAO5*), and one *RCCR* (*BvRCCR*) (Table S1). The theoretical pI and molecular weight ranged from 4.84 (HuRCCR1 and HuRCCR2) to 10.08 (HuSGR2), and from 11.8 kDa (HuSGR3) to 64.45 kDa (HuPAO1), respectively (Table S1). Indeed, most NYCs exhibited higher pI values than other chlorophyll degradation genes, whereas RCCRs showed lower theoretical pI values. Furthermore, multiple SGRs had lower molecular weights, whereas PAOs had higher molecular weights.

### 3.3. Genomic Location of Chlorophyll Degradation Genes

**Figure 2:**
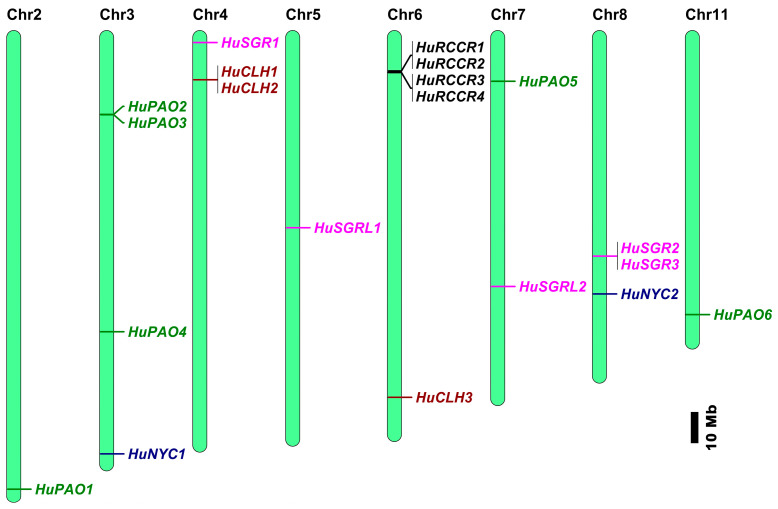
Distribution of chlorophyll degradation genes on the pitaya chromosomes. Gene names are shown on the right of each chromosome. Gene positions and the size of each chromosome can be estimated using the scale on the right of the figure, which indicates 10 megabases (Mb).

Based on chromosomal location analyses, twenty chlorophyll degradation-related genes were unequally distributed across eight chromosomes in the pitaya genome, with no genes located on chromosomes 1, 9, and 10 (Figure 2). Among them, *HuNYC1* and *HuNYC2* were located on chromosomes 3 and 8, respectively. *HuCLHs* were located on chromosomes 4 and 6, *HuSGRs* were on chromosomes 4, 5, 7, and 8, *HuPAOs* were on 2, 3, 7, and 11, and *HuRCCRs* were only detected on chromosome 6. Notably, many chlorophyll degradation genes formed gene cluster structures in pitaya chromosomes, including *HuPAO2* and *HuPAO3* in chromosome 3, *HuCLH1* and *HuCLH2* in chromosome 4, *HuRCCR1*, *HuRCCR2*, *HuRCCR3*, and *HuRCCR4* in chromosome 6, as well as *HuSGR2* and *HuSGR3* in chromosome 8.

### 3.4. Phylogenetic, Motif Composition, and Conserved Domain Analyses

**Figure 3:**
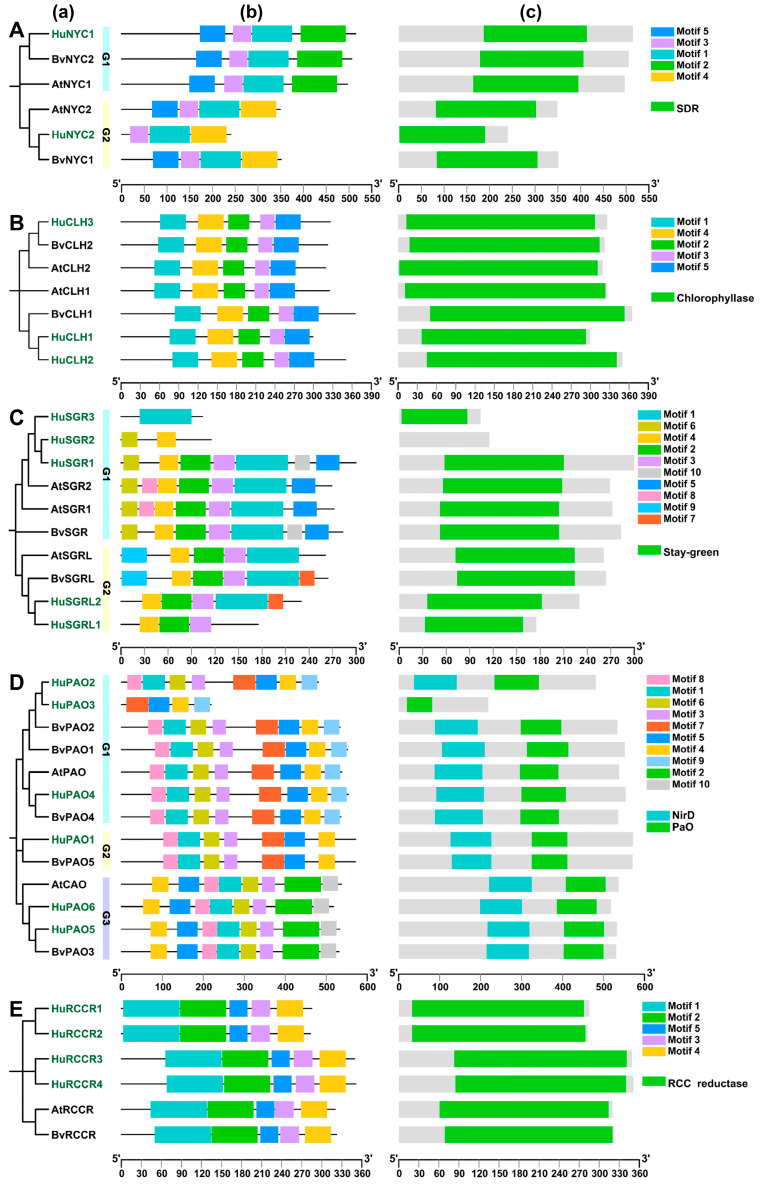
Phylogenetic tree (**a**), motifs composition (**b**), and conserved domains (**c**) of NYC (**A**), CLH (**B**), SGR (**C**), PAO (**D**), and RCCR (**E**) proteins from pitaya, beet, and Arabidopsis. The phylogenetic trees were constructed based on their amino acid sequences with 1000 bootstrap. G1, G2, and G3 refer to groups 1, 2, and 3, respectively. Amino acid sequences of these genes were provided in Table S1. The green fonts in these trees indicate proteins from pitaya.

To explore the putative function of chlorophyll degradation genes, we analyzed the phylogenetic relationships, motif composition, and conserved domains of all NYC, CLH, SGR, PAO, and RCCR proteins from pitaya, beet, and Arabidopsis. For CLHs, all of them contained the same motif order of 1, 4, 2, 3, and 5, which collectively constituted the chlorophyllase domain (Figure 3B). All RCCR proteins possessed a motif order of 1, 2, 5, 3, and 4, which formed the RCCR reductase domain (Figure 3E).

NYC amino acid sequences had conserved motifs 1, 3, and 5, which formed the short-chain dehydrogenases/reductases (SDR) domain (Figure 3A). As motif 2 only exists in HuNYC1, BvNYC2, and AtNYC1 proteins, while motif 4 only exists in the HuNYC2, BvNYC1, and AtNYC2 proteins, they gather into two distinctive groups (G1 and G2), and the sequence lengths of G1 proteins were longer than G2 proteins.

All SGR superfamily members had the stay-green domain, which was constituted by motifs 1, 2, 3, and 4, except that the HuSGR2, HuSGR3, and HuSGRL1 proteins contained partial motifs, and no stay-green domain was detected in HuSGR2 (Figure 3C). SGR superfamily members were classified into two groups, termed G1 (SGR subfamily) and G2 (SGRL subfamily) groups, because these two distinctive groups had different motif composition in the N- and C-terminal. In detail, motifs 6 and 8 at the N-terminal and motifs 5 and 10 at the C-terminal were detected in the SGR subfamily, while motif 9 at the N-terminal and motif 7 at the C-terminal were found in the SGRL subfamily. Interestingly, the amino acid lengths of HuSGR2 and HuSGR3 were significantly shorter than those of other normal SGR proteins, indicating that they were incomplete SGR sequences. In addition, HuSGR2 and HuSGR3 share a close distance on chromosome 8, and with the order of motifs 6 and 4 of HuSGR2 and motif 1 of HuSGR3, they probably function together as a normal SGR protein.

As shown in Figure 3D, PAO family members were clustered into three distinctive groups where G1 proteins formed a motif order of 8, 1, 6, 3, 7, 5, 4, and 9, G2 sequences had a motif order 8, 1, 6, 3, 7, 5, and 4, and G3 sequences had an order of 4, 5, 8, 1, 6, 3, 2, and 10. All PAO proteins contained motifs 1, 3, 4, 5, 6, and 8, except that HuPAO3 had only partials. Notably, motifs 5 and 4 were located at the C-terminal of G1 and G2 proteins, but were arranged in reverse order at the N-terminal of G3 proteins. Moreover, motifs 1, 6, and 8 formed the NirD domain, while motifs 5 and 7 of G1 and G2 sequences, or motif 2 of G3 sequences, formed the PAO domain.

### 3.5. Synteny Analysis of Chlorophyll Degradation Genes

**Figure 4:**
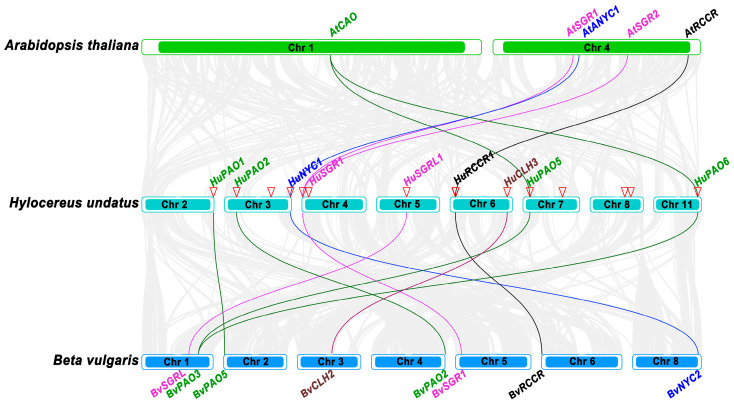
Synteny analyses of chlorophyll degradation genes from pitaya, beet, and Arabidopsis. Gray lines in the background indicate collinear blocks between genomes, while other colorful lines highlight syntenic gene pairs involved in chlorophyll degradation. Red triangles indicate the location of chlorophyll degradation genes in pitaya chromosomes.

To examine the evolutionary patterns of chlorophyll degradation-related genes, a comparative synteny analysis of these genes among pitaya (*H. undatus*), beet (*B. vulgaris*), and Arabidopsis (*A. thaliana*) species was conducted. The results demonstrated that nine collinear gene pairs were detected between pitaya and beet, and six between pitaya and Arabidopsis (Table S4 and Figure 4). More gene pairs were detected between pitaya and beet rather than between pitaya and Arabidopsis, which was consistent with the fact that both pitaya and beet belong to the Caryophyllales order and share a closer evolutionary distance between them. Among these gene pairs, *HuNYC1* showed collinearity with *AtNYC1* and *BvNYC2*, while *HuRCCR1* was collinear with *AtRCCR* and *BvRCCR*. Besides, *HuPAO5* and *HuPAO6* presented a collinearity relationship with *AtCAO* and *BvPAO3*, while *HuSGR1* was associated with *AtSGR1*, *AtSGR2*, and *BvSGR*. Aside from that, *HuCLH3*, *HuSGRL1*, *HuPAO1*, and *HuPAO2* only share collinearity with genes from beet — *BvCLH2*, *BvSGRL*, *BvPAO5*, and *BvPAO2*, respectively. Thus, the collinearity among these genes was consistent with the results of their phylogenetic tree (Figure 3a and Figure 4).

### 3.6. Expression Analyses of Chlorophyll Degradation Pathway Genes

**Figure 5:**
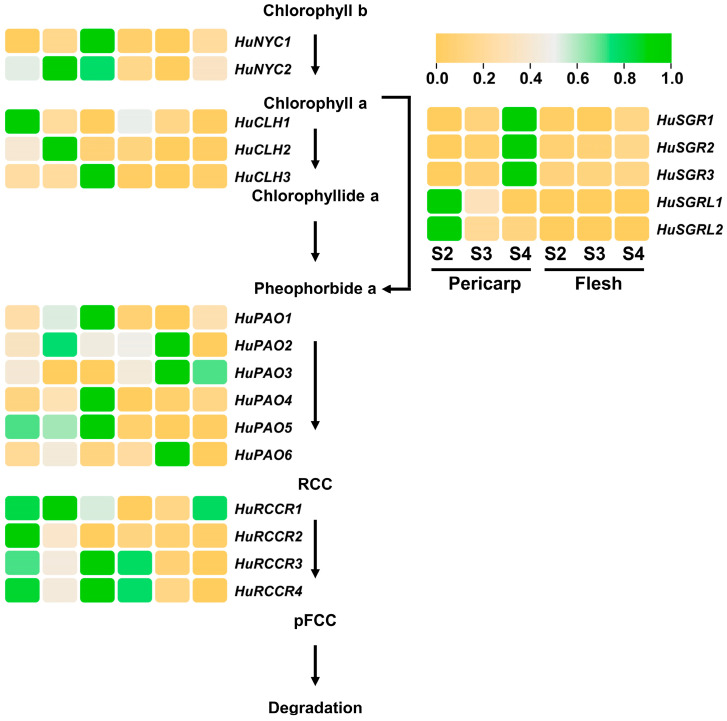
Transcript abundance of candidate genes involved in the chlorophyll degradation pathway in the pitaya peels. RCC, red chlorophyll catabolite; pFCC, primary fluorescent chlorophyll catabolite.

As the fruit matures, chlorophyll degradation naturally occurs with betalain accumulation in pitaya peels. Thus, the normal breakdown process of chlorophyll is vital for the coloring of pitaya fruits. To analyze the expression patterns of chlorophyll degradation pathway genes, the FPKM values of these genes at three different developmental stages of ‘JD1’ pitaya peels and fleshes were obtained from the transcriptome data (Table S2 and Figure 5). The results showed that *HuCLH1*, *HuSGRL1*, *HuSGRL2*, and *HuRCCR2* exhibited a downward trend during the developmental stages of pitaya peels, while showing relatively low expression levels in pitaya fleshes. On the contrary, seven genes (*HuNYC1*, *HuCLH3*, *HuSGR1*, *HuSGR2*, *HuSGR3*, *HuPAO1*, and *HuPAO4*) kept an upward trend in pitaya peels, while demonstrating relatively low expression levels in pitaya fleshes. These results suggested that these eleven genes were mainly expressed in pitaya peels and may be critical to the chlorophyll degradation process in pitaya peels.

**Figure 6:**
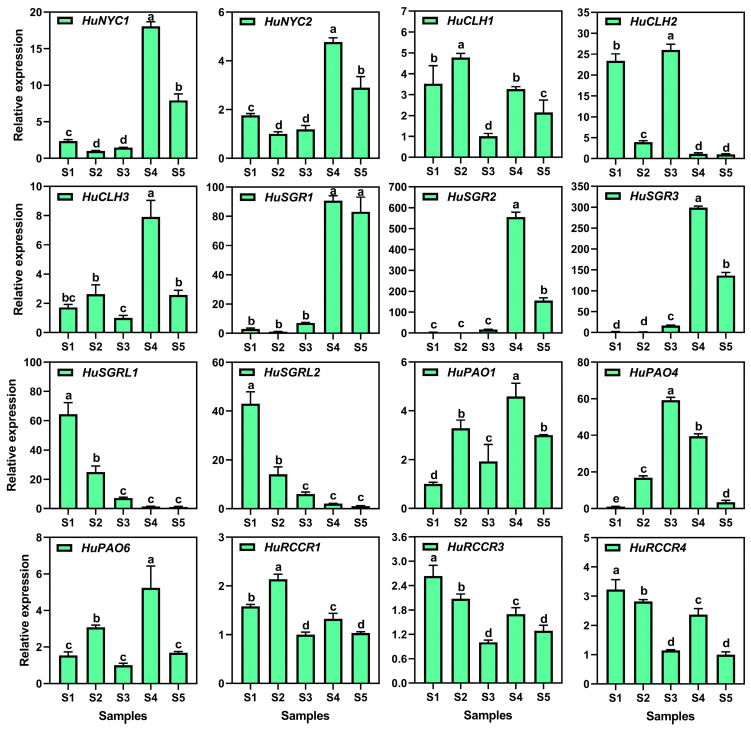
RT-qPCR analysis of chlorophyll degradation genes during the developmental stages of pitaya peels. Each presented value represents the mean ± SD with three technical repetitions. Lowercase indicates comparison between groups using one-way ANOVA (Duncan test, *p* < 0.01).

To further confirm candidate genes related to the chlorophyll degradation pathway, RT-qPCR assays were performed during the fruit maturation of pitaya peels (Figure 6). A total of 16 chlorophyll degradation-related genes were identified, of which *HuSGRL1* and *HuSGRL2* maintain a downward trend during pitaya peel maturation, consistent with the FPKM values and chlorophyll content changes. Remarkably, *HuNYC1*, *HuNYC2*, *HuCLH3*, *HuSGR1*, *HuSGR2*, *HuSGR3*, *HuPAO1*, *HuPAO4*, and *HuPAO6* were first upward and then downward, and highly expressed at S4, except that *HuPAO4* was highly expressed at S3. Their expression patterns were in line with the phenotype that chlorophyll was replaced by betalains at S3 and S4. Notably, the relative expression levels of *HuSGR2* and *HuSGR3* were significantly higher in the red peel stages, suggesting that they play important roles in the chlorophyll degradation process of pitaya peels.

### 3.7. Sequence Analyses of HuSGRs

**Figure 7:**
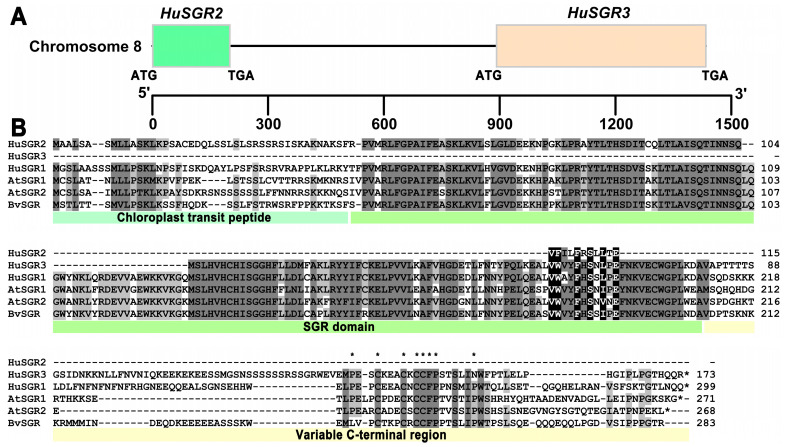
Sequence analyses of pitaya *HuSGRs*. (**A**) Distribution of *HuSGR2* and *HuSGR3* on chromosome 8. The line between two genes indicates a DNA fragment. (**B**) Multiple sequence alignment of *SGRs*. Conserved SGR domain, chloroplast transit peptide, and variable C-terminal region are labeled in different colors. A cysteine-rich motif (P-x_3_-C-x_3_-C-x-C_2_-F-P-x_5_-P) is marked with black asterisks.

SGR is a vital protein that catalyzes the first step of the chlorophyll degradation pathway in green plants. On the basis of expression analyses, we preliminarily identified that *HuSGR2* and *HuSGR3* were vital for chlorophyll degradation in pitaya peels (Figure 6). Based on their chromosome locations, *HuSGR2* and *HuSGR3* can form a gene cluster with the same transcription direction, separated by 690 bp on pitaya chromosome 8 (Figure 7A). Then, the CDSs of *HuSGR2* and *HuSGR3* were successfully cloned from pitaya, obtaining 348 and 522 bp, respectively. Compared with *HuSGR1*, which consisted of chloroplast transit peptide, SGR domain, and variable C-terminal region, *HuSGR2* only contained chloroplast transit peptide and partial SGR domain, while *HuSGR3* had partial SGR domain and variable C-terminal region (Figure 7B). In addition, the cysteine-rich motif (P-x_3_-C-x_3_-C-x-C_2_-F-P-x_5_-P) was conserved in the variable C-terminal region of *HuSGR1* and *HuSGR3*. Collectively, *HuSGR2* and *HuSGR3* might function together as a complete *SGR* in the chlorophyll degradation pathway of pitaya peels.

### 3.8. Transient Overexpression of HuSGR2 and HuSGR3 in N. benthamiana Leaves

**Figure 8:**
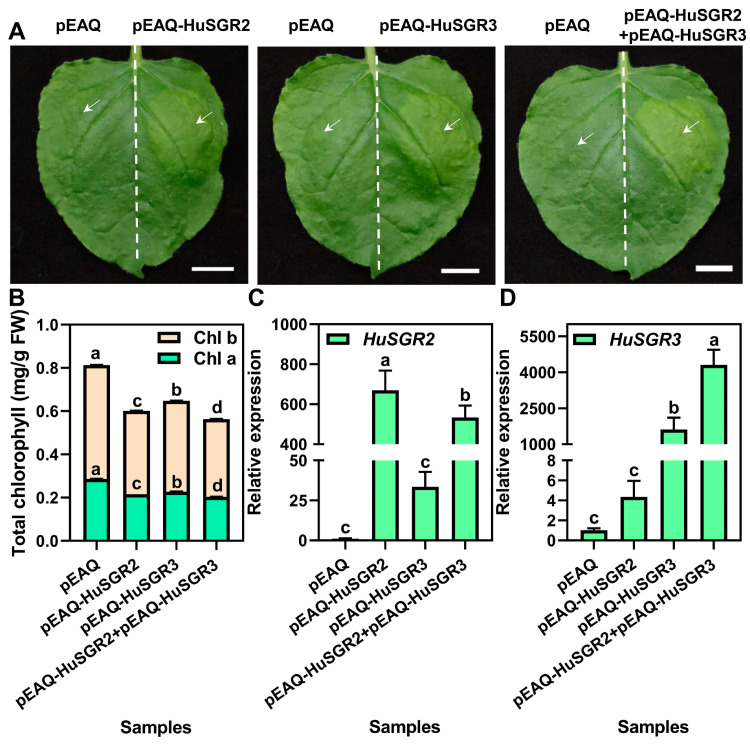
Transient overexpression of *HuSGR2* and *HuSGR3* in *N. benthamiana* leaves. (**A**) The images of *N. benthamiana* leaves after injection. Arrows indicate the injection site. Bars indicate 1 cm. (**B**) Total chlorophyll contents of injected samples. (**C**,**D**) The RT-qPCR analyses of *HuSGR2* (C) and *HuSGR3* (D) in the injected samples. Each presented value represents the mean ± SD with three biological repetitions. Lowercase indicates the comparison between groups using one-way ANOVA (Duncan test, *p* < 0.01).

To clarify the roles of *HuSGR2* and *HuSGR3*, transient overexpression assays were conducted in the *N. benthamiana* leaves. The results demonstrated that either infiltrating pEAQ-HuSGR2 or pEAQ-HuSGR3 alone led to a yellowing phenotype in tobacco leaves, whereas the empty control (pEAQ) remained green. Moreover, co-injection of pEAQ-HuSGR2 and pEAQ-HuSGR3 resulted in a more yellowing phenotype (Figure 8A). The chlorophyll content analysis indicated that overexpression of *HuSGR2* or *HuSGR3* alone resulted in a significant decrease of chlorophyll *a* and *b* contents in tobacco leaves when compared to the empty control, and a more remarkable decrease in chlorophyll *a* and *b* contents was detected when *HuSGR2* and *HuSGR3* were co-overexpressed in leaves (Figure 8B). The RT-qPCR analysis showed that overexpression of *HuSGR2* or *HuSGR3* alone strongly increased their own transcript levels, but had little effect on each other’s expressions, while notably promoting *HuSGR3* expression levels after co-overexpressing them (Figure 8C,D). These results suggested that *HuSGR2* and *HuSGR3* were involved in the chlorophyll degradation process, and accelerated chlorophyll degradation when working together.

## 4. Discussion

The peel coloration is a complex physiological process that involves multiple dynamic changes, including chlorophyll degradation, pigment biosynthesis, and responses to abiotic stress. During the fruit ripening, chlorophyll contents demonstrated a downward trend in ‘JD1’ pitaya peels, which was consistent with the other pitaya varieties ‘Guanhuabai’, ‘Guanhuahongfen’, and ‘Guanhuahong’ [19]. Thus, the normal de-greening of peels is crucial for healthy fruit development and the formation of the characteristic fruit color. However, genes from the chlorophyll degradation pathway have not been systematically explored in the pitaya genome. Herein, we identified two *NYCs*, three *CLHs*, five *SGRs*, six *PAOs*, and four *RCCRs* distributed in eight chromosomes (Table S1). Pitaya and beet are Caryophyllales species, sharing a closer evolutionary distance than the model plant Arabidopsis [26]. In order to better understand the conserved domains and the evolutionary and synteny relationships of pitaya chlorophyll degradation genes, we also identified two *NYCs*, two *CLHs*, two *SGRs*, five *PAOs*, and one *RCCR* in the beet genome and two *NYCs*, two *CLHs*, three *SGRs*, two *PAOs*, and one *RCCR* in the Arabidopsis genome (Table S1). The number of pitaya chlorophyll degradation genes was greater than those in beet and Arabidopsis, which is consistent with their genome sizes, where pitaya (~1.4 Gb) was much larger than beet and Arabidopsis [26,27,28].

Genome-wide identification and characterization of a gene family hold pivotal roles in exploring various relationships among their members, including phylogenetics, collinearity, as well as conserved domains and motifs composition. For *CLH* and *RCCR* gene family members from pitaya, beet, and Arabidopsis, the conserved motifs were identical and consisted of the chlorophyllase and RCC reductase domains, respectively. Among them, *HuCLH3* only shared a collinearity relationship with *BvCLH2*, while *HuRCCR1* shared a collinearity relationship with *BvRCCR* and *AtRCCR*. The common conserved motifs of *NYC*, *SGR*, and *PAO* gene families made up SDR, stay-green, and PaO domains, respectively, while their distinctive conserved motifs led their members to cluster into two, two, and three subfamilies. In particular, the G1 members of *NYC*, G1 of *SGR*, and G3 of *PAO* gene families shared synteny, while G2 members of *SGR* and *PAO* only shared a synteny relationship between pitaya and beet species. More gene pairs among chlorophyll degradation genes were detected between pitaya and beet than Arabidopsis, which was consistent with the synteny results of pitaya WRKY and R2R3-MYB gene family members [25,29].

Recently, the expression levels of 24 chlorophyll degradation genes were investigated with the transcriptome data of the young and coloring stages of ‘Honghuaqinglong’, ‘Guanhuabai’, and ‘Dahong’ pitaya peels, suggesting that most genes were more highly expressed in red peels than in green peels [30]. Herein, for ‘JD1’ pitaya peels and fleshes, the transcriptome and RT-qPCR analyses proved that *HuNYCs*, *HuCLHs*, *HuSGRs*, *HuPAOs*, and *HuRCCRs* were more highly expressed in pitaya peels rather than flesh, and *HuSGR1*, *HuSGR2*, and *HuSGR3* demonstrated a significantly boosted expression in red peels. Further sequence analysis showed that *HuSGR1* consisted of a full chloroplast transit peptide, SGR domain, and variable C-terminal region, which has been verified to play a vital role in pitaya chlorophyll degradation [30]. Notably, *HuSGR2* had the N-terminal conserved domain of *HuSGR1*, while *HuSGR3* had the C-terminal conserved domain of *HuSGR1*, and they are clustered together on chromosome 8, separated by 690 bp. Given that *HuSGR2* and *HuSGR3* have the same expression patterns, complementary sequence features, and closer physical distance, we supposed that *HuSGR2* and *HuSGR3* are also involved in pitaya chlorophyll degradation and probably function together as a gene cluster.

A great deal of studies indicated that tobacco leaves were an ideal material for investigating the functional roles of chlorophyll degradation genes [4,11,31]. By this method, the cysteine-rich motif of *AtSGR1* at the C-terminus was verified to be indispensable for promoting chlorophyll degradation [32]. In pitaya, the transient overexpression of *HuSGR2* and *HuSGR3* in tobacco leaves suggested that both alone play important roles in chlorophyll degradation, and their co-expression resulted in a more significant reduction in chlorophyll content and a boosted expression of *HuSGR3*. The functional roles of *HuSGR2* and *HuSGR3* that participated in the chlorophyll degradation of pitaya peels were preliminarily analyzed by a tobacco transient expression assay, whereas their roles should be further verified with pitaya genetic transformation and gene editing techniques. Moreover, whether they can form a complex, are co-regulated by specific transcription factors, or work together with other modes, also requires more experiments to clarify. In litchi and peach fruits, *SGR* serves as an indirect regulator of genes involved in chlorophyll degradation and anthocyanin biosynthesis, and was regulated by NAC transcription factors [4,31]. Studies have also reported that NAC, MADS, and WRKY regulate both chlorophyll degradation and carotenoid biosynthesis by targeting *SGR* in citrus fruits [33,34,35,36]. Both chlorophyll degradation and betalain biosynthesis processes simultaneously occurred during pitaya peel ripening, and MYB, bHLH, WRKY, and SPL transcription factors have been characterized to participate in pitaya betalain biosynthesis [29,37,38,39], but more research is needed to investigate whether these transcription factors also have a role in regulating the expression levels of *HuSGR2* and *HuSGR3*. The molecular regulatory network governing those two metabolic pathways in pitaya peel coloration remains to be further explored.

## 5. Conclusions

In summary, chlorophylls gradually degraded during the fruit development and maturation of pitaya peels. Based on pitaya genomes, twenty chlorophyll degradation genes were identified and distributed across eight chromosomes. The conserved domains and motifs of *HuCLHs* and *HuRCCRs* were identical across pitaya, beet, and Arabidopsis species, whereas *HuNYCs*, *HuSGRs*, and *HuPAOs* were divided into two or three groups with distinct motif compositions. Additionally, more collinear gene pairs of chlorophyll degradation genes were found between pitaya and beet than between pitaya and Arabidopsis. The expression patterns analysis showed that *HuSGR2* and *HuSGR3* were much more highly expressed in the red peel samples with an upward trend during fruit maturation. Further sequences of *HuSGR2* and *HuSGR3* suggested that they respectively contained the N- and C-terminal conserved domains of a complete *SGR* named *HuSGR1*. Overexpression of *HuSGR2* or *HuSGR3* separately led to significant chlorophyll degradation, while co-expression resulted in more remarkable chlorophyll degradation. Our work gives a genome-wide analysis of the chlorophyll degradation genes in pitaya fruits, clarifies the functional roles of *HuSGR2* and *HuSGR3* in the chlorophyll degradation, and provides new insights into the pitaya peel pigmentation mechanisms.

## Data Availability

The original contributions presented in this study are included in the article or Supplementary Materials. Further inquiries can be directed to the corresponding author.

## Supplementary Materials

The following supporting information can be downloaded at: https://www.mdpi.com/article/10.3390/genes17040427/s1, Table S1: Amino acid composition and physical characteristics of the pitaya chlorophyll degradation genes. Table S2: FPKM value of candidate genes related to chlorophyll degradation pathway. Table S3: The primers used in this study. Table S4: Synteny relationships of chlorophyll degradation genes among pitaya, beet, and Arabidopsis species.
